# Validation of expected objective performance indicators in surgical data analysis

**DOI:** 10.1371/journal.pone.0355276

**Published:** 2026-08-07

**Authors:** Ye Li, Sreeram Kamabattula, Kiran Bhattacharyya, Max Berniker

**Affiliations:** 1 Advanced Product Development, Intuitive Surgical, Inc., Peachtree Corners, Georgia, United States of America; 2 Advanced Product Development, Intuitive Surgical, Inc., Sunnyvale, California, United States of America; Peking University, CHINA

## Abstract

Objective Performance Indicators (OPIs) quantify surgical activity by measuring critical aspects of a case (e.g., surgeon movements, interaction forces, etc.) at clinically meaningful steps. They are particularly useful in robotic-assisted surgery, where the information required to compute them can be harvested automatically, and early studies have found that they help quantify surgeon skill. However, since surgical procedures can be highly variable and the boundaries between surgical steps often subject to interpretation, OPIs become similarly variable. Yet, OPIs should be insensitive to this variability to remain informative and unambiguous. Previously we proposed a method for computing expected OPI values in a probabilistic framework that has theoretical support for being robust to this variability. Here we validate this method and compare it against the conventional approach on a large clinical dataset. Using 5408 annotated clinical steps from 1,016 clinical cases across seven procedure types, we performed an extensive comparison of this new method alongside the conventional approach. A series of analyses compared the resulting OPI values focusing on potential biases and the variability of their values in the presence of annotator noise. We found that the probabilistic method resulted in OPI values that were less often biased than their conventional OPI counterparts (49.37%, and 56.63% respectively), and the bias magnitudes were smaller. Finally, we found that the probabilistic method resulted in significantly less variable OPI values in 40.56% of comparison, whereas 8.52% showed the opposite. Our analysis suggests that the expected OPIs are more robust to annotator variability, with less bias and smaller variance. We suggest that this probabilistic approach holds promise for the computational analysis, and perhaps the conceptual interpretation, of surgical data.

## Introduction

Robotic assisted surgery (RAS) is growing in prominence and prevalence. The number of cases performed each year with RAS is growing at an exponential rate [1], and the types of procedure offered are expanding [2,3]. Importantly, each RAS procedure yields a wealth of data as a matter of course (e.g., video, surgeon motor behavior, tool kinematics, forces, etc.), providing surgeons and scientists with the unique opportunity to analyze surgeries in precise and quantifiable terms. Objective performance indicators (OPIs) are one such means of quantifying surgery.

An OPI is a metric to analyze surgical activity during a specific time window. For example, how quickly and far tools were moved, or the forces used to interact with tissue, or cauterization rates, during a particular window of activity, are all features that can be quantified through OPIs. The windows of activity they examine can be defined with different levels of granularity, such as an entire case, a phase, or what we focus on here, a single surgical step. As evidence of their importance, connections between OPIs measured during critical categories of steps and surgeon skills have already been made [4,5]. Choosing not only which feature to measure, but which step to measure it, may prove crucial for OPI analyses.

Unfortunately, unambiguously identifying steps can prove particularly challenging. For a given surgical case, various techniques, surgeons’ preferred strategies, and patients’ unique anatomies all increase the variability of surgical steps. In addition, annotations are usually made postoperative, without the benefit of the surgeon’s direct input. As such, the boundaries (start and stop times) of steps can be ambiguous and subject to interpretation. This variability, in turn, renders the OPIs variable and potentially biased, limiting their precision in evaluating surgical performance. A method for computing OPIs that is robust to this annotation variability is paramount.

We previously proposed a probabilistic framework for computing OPIs [6]. Rather than computing OPIs with “hard” temporal boundaries (i.e., discrete points in time for the start and stop of tasks), we described annotations as random samples from an underlying distribution and computed the resulting expected OPI value. We demonstrated how the expected OPIs, approximated using a finite sample of annotations, were more accurate and precise (with smaller biases and variances) than the conventional hard-boundary method.

The aim of this study is to verify those theoretical benefits on a rich, real-world dataset. We gathered data from seven procedure types and over a thousand surgical cases. Accompanying each case were the annotations, kinematics, and system event data necessary to compute OPI values. For each annotation we examined five different OPIs, computing both their hard-boundary and expected values. Using empirical estimates of annotation variability, we found that the expected OPI values were more accurate and less variable, verifying the theoretical benefits of the probabilistic approach. We suggest it may be helpful to think of OPIs as random variables that must be estimated.

## Materials and methods

### Data collection

In this study, we retrospectively collected data from 1,016 de-identified surgical cases performed using the da Vinci^®^ Xi Surgical System. These cases included seven procedure types: lobectomy (N = 238), ventral hernia (N = 76), hysterectomy (N = 137), low anterior resection (N = 56), radical prostatectomy (N = 54), inguinal hernia (N = 211), and cholecystectomy (N = 244). For each case, we captured the tool kinematics (position and orientation) of the system’s four robotic arms, along with hand and camera clutch events, and energy application events (occurring during cauterization).

Surgical procedures can be broken into a series of clinically relevant, mutually exclusive steps. Each procedure type has unique surgical steps based on an ontology derived from consensus recommendations made by surgeons [7], with detailed methodology described elsewhere [8]. For some procedure types, the parameters which defined the start and stop times of steps were modified early in our data collection process to improve annotator alignment.

All annotators first completed a standardized training and calibration process with clinical oversight provided by a trained surgeon, until annotations were within five seconds of agreement. Teams of four to seven annotators were assigned to each procedure type. Following calibration, each case was annotated by a single trained annotator, who identified the start and stop times for the case’s surgical steps.

Retrospective system data collected during surgical procedures were analyzed under an approved IRB protocol (IRB tracking number: 20182083, WCG Clinical), with all patient identifiers removed prior to analysis. De-identified raw system data for this study were accessed between 01/09/2022 and 16/12/2024. Consent form was obtained from surgeons and patients before data was collected.

### Objective performance indicators

Objective performance indicators (OPIs) computed over surgical steps can quantify any number of surgeon behaviors or techniques. Typical OPIs include the duration of a step, or the distance travelled by an instrument [9]. These OPIs are usually computed via integrals, or in discrete-time, sums.

For example, suppose ${OPI}_{f}$ quantifies some function, f(t), integrated from $t_{start}$ to $t_{stop}.$ Assume f(t) is defined over a set of equally spaced discrete values in time (with a step size, ∆), $\left[t_{\text{1}},t_{\text{2}},t_{\text{3}},\ldots ,t_{N}\right]$. Then ${OPI}_{f}$, from start time, $t_{start}=t_{i}$ to stop time, $t_{stop}=t_{j}$, is computed as:


$$\begin{array}{c} {OPI}_{f}=\sum_{k=\text{0}}^{m}f\left(t_{i}+k\Delta \right)\Delta =\sum_{k=i}^{j}f(t_{k})\Delta \end{array}$$ (1)

where $m=(t_{j}-t_{i})/\Delta$. In this study, we refer to OPIs computed by this method as the “hard-boundary” OPIs.

As an alternative, consider the scenario where the start and stop times of a step are not known, but rather we have a belief, or joint distribution, over start and stop times, $P(t_{start},t_{stop})$. The OPI is now a random variable. To compute the expected OPI value we begin with the probability of a step. That is, $P_{step}(t)$ is the probability of a step at time t, implying $t_{start}\leq t$ and $t_{stop}>t$:


$$\begin{array}{c} P_{step}\left(t=t_{k}\right)=P\left(t_{start}\leq t_{k},t_{stop}\geq t_{k}\right)=\sum_{i=\text{1}}^{k}\sum_{j=k+\text{1}}^{N}P(t_{i},t_{j}) \end{array}$$ (2)

The expected OPI value, ${\overset{―}{OPI}}_{f}$ can be found as:


$$\begin{array}{c} {\overset{―}{OPI}}_{f}=\sum_{k=\text{1}}^{N}f(t_{k})P_{step}(t_{k})\Delta \end{array}$$ (3)

This is our best estimate of ${OPI}_{f}$ when it is a random and uncertain variable. We refer to OPIs computed by this method as “expected” OPIs.

Prior to this investigation, an internal study was conducted to quantify the uncertainty associated with human annotation of surgical steps. Multiple annotators independently annotated the same cases. Using these replicate annotations, we estimated the variance of start and stop annotations and calculated the corresponding standard deviation, which was approximately two seconds. We observed that annotation variability did not differ substantially across steps or procedure types. Based on these findings, we assumed the joint distribution of annotations, $P(t_{start},t_{stop})$, could be approximated with two independent normal distributions, both with standard deviations of two seconds. In this study we chose to limit our analysis to surgical steps that had only a single annotated instance per case, since two or more instances of the same step in quick succession could invalidate our normality assumption for $P(t_{start},t_{stop})$ (e.g., if the stop of one instance was less than two seconds before the start of the next instance). For a complete list of surgical steps analyzed see S1 Table.

We computed five different OPIs for the surgical steps in this study, using the two methods described above. These OPIs and their definitions are as follows: (1) tool path length: the total path length traveled by all instruments during a given surgical step, (2) angular path length: the total endowrist angular path length traveled by all instruments during a given surgical step, (3) camera clutch rate: the average rate of endoscope clutches performed during the step, (4) energy use rate: the average rate of energy pedal presses during the step, and (5) hand clutch rate: the average rate of hand controller clutches performed during the step.

### Statistical analyses

To verify that the probabilistic framework for computing OPIs is superior to the conventional hard-boundary method, we tested two aspects: 1) whether the expected OPI was more accurate than the hard-boundary OPI, and 2) whether the expected OPI was more robust to annotator variability. To do so we performed the following resampling process. For each single-instance step, we used the annotator derived start and stop times to compute “nominal” hard-boundary and expected OPI values. These nominal values were used as reference OPI values for subsequent comparisons. Then, using a normal distribution centered on these nominal annotations we resampled 500 start and stop time pairs. We used a standard deviation of two seconds, based on an earlier study of the variability of annotations among skilled annotators. For edge instances where either the step start or stop time was the beginning or the end of the procedure respectively, we adjusted the resampling process and only resampled the free unconstrained boundary. For each resampled pair, the five OPIs were calculated using the two methods.

To compare accuracy, we evaluated how closely the randomly sampled OPI values matched their nominal values, and how large the sample bias was. One-sample t-tests were performed to determine whether the sampled OPIs were significantly different from their respective nominal values. We also compared the OPI values from two methods directly, using two-sample t-tests. To evaluate the sample bias, we normalized the mean bias with the nominal OPI values. Then we compared the normalized bias of two OPI methods by the Wilcoxon signed-rank test.

To assess robustness, we examined whether either method produced sample sets with a narrower range of values. We used Levene’s test to evaluate differences in sample variances between the two methods. Similar to the analyses of bias, we also computed a normalized standard deviation using the nominal OPI values. Then we compared the normalized standard deviation of two OPI methods by the Wilcoxon signed-rank test.

The percentage of steps showing significant differences in biases and variances provides insight into the extent to which OPI values may differ when replacing the conventional hard-boundary approach with the novel probabilistic approach. For all tests a significance level of α = 0.05 was applied.

## Results

Data from 1,016 surgical cases spanning seven procedure types was collected. In total, 16,124 annotated step instances were identified across all cases. Of these, 10,393 step instances (64.46%) were excluded because they occurred multiple times within a case. The proportion of excluded multi-instance steps varied across procedure types, ranging from 34.6% to 78.4%, reflecting differences in inherent workflow structure and the frequency of iterative steps within certain procedures. 323 additional step instances were excluded due to data quality issues. The remaining 5,408 single-instance surgical steps (ranging from 1 to 14 per case, mean = 5) were included in the analysis. Five nominal OPIs (instrument path length, endowrist angular length, camera clutch rate, energy use rate and hand clutch rate) were computed for each step, along with paired, randomly sampled sets of hard-boundary and expected OPI values. In total this yielded 27,040 paired OPIs for comparison and analysis.

To provide some numerical context for the OPIs, the means for the two methods are displayed below (see Table 1). These are averaged across all steps and cases within each procedure type, so no detailed conclusions should be drawn, but rather the values are displayed to convey their relative magnitudes and their variability. Note that while the OPI values do vary considerably (in both value and nature), their values are relatively similar between methods. This demonstrates that the distinctions between the two methods require a careful analysis.

**Table 1:** Mean values of hard-boundary and expected OPIs across steps of each procedure type.

Procedure type	OPI (unit)	*Mean*_*HB*_	*Mean*_*Expected*_
Lobectomy *N*_*case*_ = 238 *N*_*task*_ = 1117	PATH (m)	6.29	6.29
	ANG (rad)	201.48	205.31
	CAM (*s*^−1^)	0.06	0.06
	ENGY (*s*^−1^)	0.16	0.16
	HAND (*s*^−1^)	0.04	0.04
Ventral Hernia *N*_*case*_ = 76 *N*_*task*_ = 292	PATH (m)	20.26	20.26
	ANG (rad)	640.06	651.86
	CAM (*s*^−1^)	0.07	0.07
	ENGY (*s*^−1^)	0.07	0.07
	HAND (*s*^−1^)	0.04	0.04
Hysterectomy *N*_*case*_ = 137 *N*_*task*_ = 1047	PATH (m)	7.85	7.85
	ANG (rad)	194.14	197.46
	CAM (*s*^−1^)	0.07	0.07
	ENGY (*s* ^−1^)	0.17	0.17
	HAND (*s*^−1^)	0.04	0.04
Low Anterior Resection *N*_*case*_ = 56 *N*_*task*_ = 377	PATH (m)	12.95	12.95
	ANG (rad)	336.86	342.95
	CAM (*s*^−1^)	0.06	0.06
	ENGY (*s*^−1^)	0.15	0.15
	HAND (*s*^−1^)	0.04	0.04
Radical Prostatectomy *N*_*case*_ = 54 *N*_*task*_ = 516	PATH (m)	12.39	12.39
	ANG (rad)	309.46	316.68
	CAM (*s*^−1^)	0.10	0.10
	ENGY (*s*^−1^)	0.15	0.15
	HAND (*s*^−1^)	0.01	0.01
Inguinal Hernia *N*_*case*_ = 211 *N*_*task*_ = 1059	PATH (m)	10.56	10.56
	ANG (rad)	262.25	266.86
	CAM (*s*^−1^)	0.07	0.07
	ENGY (*s*^−1^)	0.07	0.07
	HAND (*s*^−1^)	0.04	0.04
Cholecystectomy *N*_*case*_ = 244 *N*_*task*_ = 1000	PATH (m)	4.63	4.63
	ANG (rad)	145.56	150.76
	CAM (*s*^−1^)	0.06	0.06
	ENGY (*s*^−1^)	0.16	0.16
	HAND (*s*^−1^)	0.05	0.05

PATH: tool path length; ANG: (endowrist) angular path length; CAM: camera clutch rate; ENGY: energy use rate; HAND: hand controller clutch rate. To compute the mean values for event OPIs (CAM, ENGY, HAND), we excluded the steps in which such events did not occur.

### Accuracy of expected OPIs

Our first comparison was in terms of accuracy, and in particular whether one approach provided OPI values that were closer to their nominal value when the annotations were noisy and uncertain. To do so we compared the nominal OPIs values with the means of their randomly resampled counterparts. First, we note that across procedure types and OPIs, many comparisons, indeed often most, are statistically distinct (see Table 2). In theory, the OPIs do not vary linearly with their annotations and adding noise to the annotations will bias their values. This result drives home how in practice, this is clearly the case as well. Regardless, relative to the hard-boundary OPIs, the mean expected OPIs were more often indistinguishable from their nominal values. Averaging across the five OPIs and seven procedure types, the percentage of steps whose sample means were significantly distinct from their nominal OPI values were 56.63% and 49.37%, for the hard-boundary and expected OPIs, respectively.

**Table 2:** Percentage of steps with significant biases OPI values.

Procedure type		PATH	ANG	CAM	ENGY	HAND
Lobectomy	HB	68.13	70.55	59.27	46.20	34.74
	Exp	45.39	53.80	62.94	48.08	36.53
	comparisons	28.38	86.21	52.19	42.35	33.66
Ventral Hernia	HB	68.49	69.52	55.48	26.48	31.16
	Exp	49.66	46.92	57.53	23.99	36.99
	comparisons	26.71	95.54	50.0	19.51	34.93
Hysterectomy	HB distinct	71.25	75.74	61.22	64.11	29.42
	Exp distinct	49.00	51.86	64.95	61.05	36.38
	comparisons	32.19	87.87	55.41	50.14	31.32
Low Anterior Resection	HB distinct	70.292	68.44	61.01	42.71	31.30
	Exp distinct	45.09	49.07	58.36	46.15	38.46
	comparisons	23.08	86.47	45.62	40.32	29.44
Radical Prostatectomy	HB distinct	69.57	69.57	73.06	47.87	19.77
	Exp distinct	45.54	46.90	71.90	43.99	27.52
	comparisons	23.64	93.22	60.08	36.82	25.19
Inguinal Hernia	HB distinct	67.42	69.22	67.61	31.10	31.16
	Exp distinct	44.19	42.97	68.18	29.77	36.83
	comparisons	24.17	93.11	57.51	24.10	30.97
Cholecystectomy	HB distinct	75.8	76.9	60.7	48.4	38.9
	Exp distinct	57.6	60.7	62.0	48.1	46.4
	comparisons	40.0	82.8	52.9	41.2	39.6

**HB**: percentage of hard-boundary computed steps with biased sample means. **Exp**: percentage of expected steps with biased sample means. **comparisons**: percentage of steps with significantly distinct hard-boundary and expected OPIs.

When comparing the kinematic OPIs (tool path length and angular path length) the expected OPIs showed a consistent and substantial improvement in accuracy across procedure types. Approximately 72.25% of hard-boundary OPI comparisons were statistically distinct, whereas the expected values were distinct in only 49.78% of comparisons. In summary, all five OPIs tested, particularly the kinematic OPIs, were more accurate using the probabilistic approach than those obtained with the hard-boundary approach.

Next, we examined the similarity of the values generated by the two approaches (Table 2, “comparisons”). The frequency and size of these differences would be indicative of hard-boundary biases. Similar to the above observation, the kinematic OPI of angular path length consistently had the largest number of distinct comparisons. When averaging across the five OPIs and seven procedure types, 48.61% of the hard-boundary and expected OPI samples were significantly distinct. These differences indicate the relative fragility of the hard-boundary approach.

Lastly, to provide more context we computed the average biases of the two approaches. Since the OPIs differ in category and magnitude we compared biases as an unsigned percentage of their nominal value (Table 3). As can be seen, the biases are all less than 10%, and the biases of the expected OPIs are smaller than their hard-boundary counterparts in every single example. We tested the significance of the biases (Wilcoxon signed-rank test) and found that in all but three scenarios the expected bias was significantly smaller than that of the hard-boundary bias.

**Table 3:** Mean normalized percent bias of hard-boundary and expected OPIs and the p-value of their comparisons (Wilcoxon signed-rank test).

Procedure type		PATH	ANG	CAM	ENGY	HAND
Lobectomy	Mean bias HB	0.7	1.1	2.4	2.0	2.4
	Mean bias Exp	0.4	0.5	0.8	0.7	0.8
	p-value	< 0.001	< 0.001	< 0.001	< 0.001	< 0.001
Ventral Hernia	Mean bias HB	0.5	0.6	1.0	1.4	1.5
	Mean bias Exp	0.2	0.2	0.4	0.4	0.5
	p-value	< 0.001	< 0.001	< 0.001	< 0.001	<0.01
Hysterectomy	Mean bias HB	1.1	1.2	3.2	3.9	2.8
	Mean bias Exp	0.7	0.6	1.2	1.2	1.0
	p-value	< 0.001	< 0.001	< 0.001	< 0.001	**0.120**
Low Anterior Resection	Mean bias HB	0.6	0.8	2.2	2.3	1.7
	Mean bias Exp	0.2	0.4	0.6	0.8	0.6
	p-value	< 0.001	< 0.001	< 0.001	< 0.001	**0.301**
Radical Prostatectomy	Mean bias HB	0.3	0.4	1.5	1.0	1.2
	Mean bias Exp	0.2	0.2	0.5	0.3	0.6
	p-value	< 0.001	< 0.001	< 0.001	< 0.001	**0.344**
Inguinal Hernia	Mean bias HB	0.8	0.7	2.5	2.5	2.1
	Mean bias Exp	0.3	0.3	0.8	0.8	0.9
	p-value	< 0.001	< 0.001	< 0.001	< 0.001	< 0.01
Cholecystectomy	Mean bias HB	4.3	5.6	3.5	2.2	3.0
	Mean bias Exp	1.5	1.5	1.2	0.8	1.1
	p-value	< 0.001	< 0.001	< 0.001	< 0.001	< 0.001

### Robustness of hard-boundary OPIs

Next, we compared the robustness of the two approaches, examining the relative effects of annotator noise on the resulting variability in OPI values (independent of their accuracy). We compared the sample variances across the seven procedure types and five OPIs (Levene’s test, see Table 4). Almost all OPIs had a greater number of smaller variance results with the probabilistic method. Angular path length was the exception, where there were a moderate amount of steps with larger expected OPI variance (23.30% of steps, relative to 20.28% of the steps with the hard-boundary approach). However, when averaging across the five OPIs, the probabilistic approach was superior. Finally, a weighted average (taking into account the differing steps per procedure) across OPIs and procedure types found that 40.56% of the steps had larger hard-boundary variance, whereas only 8.52% of the steps had larger expected variances.

**Table 4:** Percentage of tasks showing higher variance in one OPI method than the other.

Procedure type		PATH	ANG	CAM	ENGY	HAND
Lobectomy	% var HB > Exp	22.98	21.36	61.28	52.26	32.04
	% var Exp > HB	12.93	23.43	0.94	1.90	2.21
Ventra Hernia	% var HB > Exp	20.89	19.52	57.54	54.31	23.51
	% var Exp > HB	15.07	24.32	1.05	2.59	2.99
Hysterectomy	% var HB > Exp	22.18	20.55	65.95	73.10	29.02
	% var Exp > HB	15.58	21.70	1.02	1.15	1.45
Low Anterior Resection	% var HB > Exp	20.95	20.42	63.98	43.16	32.44
	% var Exp > HB	15.65	23.34	1.15	3.16	1.67
Radical Prostatectomy	% var HB > Exp	17.32	17.28	78.94	55.17	17.07
	% var Exp > HB	10.31	18.64	0.00	1.48	5.18
Inguinal Hernia	% var HB > Exp	19.47	13.04	71.14	64.81	27.00
	% var Exp > HB	13.04	22.68	0.39	0.86	2.83
Cholecystectomy	% var HB > Exp	32.3	28.2	68.95	70.71	41.42
	% var Exp > HB	18.4	27.6	0.58	1.21	2.55

% var HB > Exp: percentage of tasks where samples show significant higher variance in hard-boundary OPIs, compared to expected OPIs. % var Exp > HB: percentage of tasks where samples show significant higher variance in expected OPIs, compared to hard-boundary OPIs. Note: for each OPI, tasks failed to conduct Levene’s test was excluded when calculating the percentage.

As with the biases, we compared the standard deviations as an unsigned percentage of their nominal OPI value (Table 5). As can be seen, the standard deviations of the expected OPIs were significantly smaller in all conditions.

**Table 5:** Mean normalized percent of standard deviation based on the OPI values and the p-value of its comparison using Wilcoxon signed-rank test.

Procedure type		PATH	ANG	CAM	ENGY	HAND
Lobectomy	Mean Var HB	3.28	3.59	5.18	5.60	5.80
	Mean Var Exp	3.09	3.23	3.55	4.22	4.26
	p-value	< 0.001	< 0.001	< 0.001	< 0.001	< 0.001
Ventral Hernia	Mean Var HB	1.83	1.97	2.48	4.11	3.52
	Mean Var Exp	1.74	1.77	1.73	3.16	2.52
	p-value	< 0.001	< 0.001	< 0.001	< 0.001	< 0.001
Hysterectomy	Mean Var HB	4.67	4.59	7.10	9.03	6.90
	Mean Var Exp	4.58	4.31	4.83	6.47	4.92
	p-value	< 0.001	< 0.001	< 0.001	< 0.001	< 0.001
Low Anterior Resection	Mean Var HB	2.38	2.75	4.16	5.79	4.06
	Mean Var Exp	2.25	2.51	2.79	3.95	3.15
	p-value	< 0.001	< 0.001	< 0.001	< 0.001	< 0.001
Radical Prostatectomy	Mean Var HB	1.72	1.78	3.03	2.62	3.12
	Mean Var Exp	1.65	1.64	2.04	2.11	2.50
	p-value	< 0.001	< 0.001	< 0.001	< 0.001	< 0.001
Inguinal Hernia	Mean Var HB	3.37	3.23	5.28	6.52	5.30
	Mean Var Exp	3.19	3.00	3.67	5.23	3.93
	p-value	< 0.001	< 0.001	< 0.001	< 0.001	< 0.001
Cholecystectomy	Mean Var HB	12.97	14.47	7.21	5.52	6.77
	Mean Var Exp	6.28	6.63	4.84	4.23	4.93
	p-value	< 0.001	< 0.001	< 0.001	< 0.001	< 0.001

## Discussion

In a previous study we proposed a probabilistic approach to OPI computation, outlining its theoretical benefits over the conventional hard-boundary method, and presenting a proof of concept on a limited data set [6]. Here our aim was to determine if these previously reported benefits withstand a rigorous examination with a rich and extensive clinical data set. We collected data from 1,016 surgical cases, across seven procedure types, and 194 step categories. In total this provided 5,584 manually annotated surgical steps. For every annotation we computed five OPIs. This provided a uniquely large dataset of clinically-derived OPIs to validate the probabilistic OPI approach.

Across all seven procedure types and 5,584 surgical steps, we observed that expected OPIs exhibit bias less frequently than the traditional hard-boundary OPIs. This was particularly the case for the kinematic OPIs of tool path length and angular path length. Furthermore, about half of the surgical steps we examined showed distinct OPI values between the hard-boundary and probabilistic approaches, highlighting the potential benefits of transitioning to a probabilistic framework for OPI computation. In terms of variance, expected OPIs consistently demonstrated an advantage across all procedure types with more steps exhibiting significantly higher variance with the hard-boundary method than vice versa. This pattern was observed for all OPIs except angular path length, suggesting that probabilistic OPIs provide more stable measurements and are less sensitive to annotation variability than conventional hard-boundary OPIs.

Probabilistic OPIs demonstrated comparable or superior performance, and adopting them can provide additional benefits as well. Consider that probabilistic OPIs do not require a change in the established methodology for annotating steps. This is crucial since human annotations are usually the most time and resource intensive element of the analysis. There is a nominal increase in computational effort, however, and either the probability of a step or the distribution of annotation times is required. Yet this effort can be viewed as an advantage since it provides a mechanism to incorporate variability and uncertainty in the annotation process. For example, annotation variability may differ across procedures, steps, and human annotators. All these distinct sources of variability can be explicitly addressed both at the level of annotation, but also in subsequent downstream computations.

Here we limited our analysis in three important respects. First, our analysis was restricted to single instance steps. There are conditions under which a surgical step might be completed with two or more distinct portions in quick succession. When the duration between instances is short relative to the variability of an annotation, modeling annotation uncertainty is more complicated. To keep the analysis straightforward we chose to ignore all multi-instance steps regardless of whether or not they violated our assumptions. In the future we hope to explore probabilistic techniques to that are robust to these conditions. Second, we assumed a fixed annotation variability (σ = 2 seconds), derived from an internal study of inter-annotator variability. We did not perform a formal sensitivity analysis to evaluate the robustness of our results with respect to this assumption. While this representative value enabled a clear demonstration of the method, future work could assess the impact of varying levels of annotation uncertainty. Finally, we have assumed annotation uncertainty is fixed and constant across all steps and procedure types. In settings where annotation uncertainty is more likely to change, for example, with complicated workflow, context-dependent uncertainty could be examined.

OPIs are promising for a number of applications such as, evaluating surgeon skill [8–12], predicting surgical outcomes [13–16], and providing directed feedback for surgeon training [17,18]. The same basic approach of addressing variability and uncertainty employed here, could be used in these downstream OPI applications. For example, rather than providing a read-out of surgical skill as a function of OPIs, a probability distribution over skill could be computed. We hope to explore possibilities like this in the future.

Identifying the beginning and ending of surgical segments is an inherently variable process, yet crucial for computing objective performance indicators (OPIs). To mitigate the influence of this variability on OPIs, we propose taking a probabilistic approach that computes their expected value. Our results suggest that this probabilistic approach holds promise for the computational analysis, and perhaps the conceptual interpretation, of surgical data.

## Supporting information

S1 Table Surgical steps included in the analysis. (DOCX)
