# Supplementary material for: Validation of expected objective performance indicators in surgical data analysis
Source: PLoS One. 2026 Aug 7;21(8):e0355276. doi: 10.1371/journal.pone.0355276 (PMC13450760; doi:10.1371/journal.pone.0355276)
Supplement: Supplementary file 1 [file pone.0355276.s001.docx]

**Supporting Information**

**S1 Table. Surgical steps included in the analysis.**

| Lobectomy | Dissection of artery  Dissection of bronchus  Dissection of vein  Divide inferior pulmonary ligament  Division of artery  Division of bronchus  Division of vein  Fissure dissection  Lymph Node Dissection  Wedge removal |
| --- | --- |
| Ventral Hernia | Closure of hernia defect  Closure of peritoneum  Excision of existing mesh  IPOM reduction of hernia  Lysis of adhesions and bowel sweep (optional)  Measurement of hernia defect  Mesh placement and fixation  TAPP peritoneal flap exploration & reduction of hernia sac  TAPP peritoneal incision |
| Hysterectomy | Bladder flap creation  Colpotomy  Dissection skeletonization and division of the uterine vessels (left side)  Dissection skeletonization and division of the uterine vessels (right side)  Lymph node dissection on the left and right side  Mobilize colon / removal of adhesions (optional)  Para-aortic lymph node dissection  Removal of left adnexa  Removal of right adnexa  Removal of the uterus  Vaginal cuff closure |
| Low anterior resection | Colorectal anastomosis  Initial exposure  Lateral to medial mobilization of sigmoid and descending colon  Ligation and division of the named pedicle  Medial to lateral mobilization of the rectum, sigmoid, and descending colon  Preparation of the distal end of the specimen for transection  Preparation of the proximal end of the specimen for transection  Splenic flexure mobilization  Total mesorectal excision  Splenic flexure mobilization  Transection of the descending colon (proximal end of specimen)  Transection of the rectum (distal end of specimen) |
| Radical prostatectomy | Anterior bladder neck dissection  Apical dissection  Drop bladder  DVC stitch  Endopelvic fascia dissection  L pedicles / nerve sparing  Lymph node dissection l  Lymph node dissection r  Posterior plane / denonvilliers anterior approach  Posterior plane / denonvilliers posterior approach  Rocco stitch  R pedicles / nerve sparing  Seminal vesicles anterior approach  Seminal vesicles posterior approach  Vesico-urethral anastomosis |
| Inguinal hernia | B/L mesh placement and fixation  Left closure of peritoneum  Left creation of peritoneal incision  Left lysis of adhesions and bowel sweep (optional)  Left measurement of hernia defect (optional)  Left mesh placement and fixation  Left peritoneal flap exploration/reduction of hernia sac  Right closure of peritoneum  Right creation of peritoneal incision  Right lysis of adhesions and bowel sweep (optional)  Right mesh placement and fixation  Right peritoneal flap exploration/reduction of hernia sac |
| Cholecystectomy | Amputation of gallbladder  Dissection of calots triangle  Dissection of gallbladder off liver bed  Hemostasis of liver bed  Initial exposure  Intentional gallbladder puncture (optional)  Ligation and division of cystic artery and cystic duct  Ligation/division of cystic artery  Ligation/division of cystic duct  Removal of adhesions (optional)  Specimen removal |

Two universal surgical steps – “Other” and “Hemostasis” – were also included in the analytical dataset. These steps are not shown here because they are not specific to any single operative phase but were consistently present across procedures.

During the formalization of the surgical‑step ontology, several step names were revised and, in some cases, multiple granular steps were combined to improve annotator consistency and alignment. To avoid confusion, only the final standardized step names are presented in this table; earlier versions and archived labels are not shown.
