# Supplementary material for: Validation of expected objective performance indicators in surgical data analysis
Source: PLoS One. 2026 Aug 7;21(8):e0355276. doi: 10.1371/journal.pone.0355276 (PMC13450760; doi:10.1371/journal.pone.0355276)
Supplement: Supplementary file 4 [file pone.0355276.s005.docx]

Dear reviewer,

We thank the Reviewer for their thoughtful and constructive evaluation of our manuscript. Below, we provide point-by-point responses to all comments. All changes have been incorporated into the revised manuscript, with corresponding sections indicated.

**Comment 1: Surgical step annotation framework**

The authors state that surgical steps were defined using an ontology informed by consensus recommendations (ref. 7). While this reference provides a strong conceptual framework supporting structured, consensus-based step annotation, additional methodological detail would improve transparency and reproducibility.

Please clarify:

- How the step definitions were developed in practice following the principles outlined in the SAGES consensus.
- Whether the same step definitions were applied consistently across all cases within each procedure type.
- How annotators were trained and calibrated to apply these step definitions.

**Response 1:** We thank the reviewer for raising these important topics. We have revised the manuscript accordingly to provide the additional methodological detail. Our responses to the specific points are provided below.

1. To improve clarity, we have added a citation to a recently published paper from our annotation team that provides a detailed description of how surgical step annotations are defined, operationalized, and applied in practice within our annotation workflow. This paper describes the construction and use of step-level annotation ontologies, the annotation process, and quality control procedures, and is now cited in the revised manuscript (see Method A. Data Collection paragraph 2).

2. Yes, for the majority of cases, the same step definitions were applied consistently. However, for a few procedure types, small adjustments to the start and stop parameters of a step (though not the definition of the step) were introduced early in the dataset construction, to improve inter-annotator alignment. From that point forward, all annotations were consistently applied to cases of that procedure type.

3. Annotators underwent a structured training and calibration process prior to contributing annotations. Training typically consisted of annotating 10–20 representative cases of the same procedure type, spanning a range of case complexity and approaches. During this phase, each trainee’s annotations were compared against a “gold-standard” reference produced by the most experienced annotator for that procedure, under surgeon oversight.

Calibration criteria required temporal alignment of step start and end times within approximately 5 seconds of the gold-standard annotations. Trainees received iterative feedback and were only authorized to annotate independently after achieving this alignment criterion. Annotators who did not meet the required agreement threshold continued training until alignment was achieved or were not assigned to that procedure type.

In addition to initial training, ongoing quality control was performed through periodic consensus reviews (e.g., quarterly or bi-quarterly), during which inter-annotator alignment was reassessed to ensure long-term consistency. All annotators followed the same training and calibration protocol, and annotation teams for each procedure typically consisted of approximately 4–7 trained annotators, with overall clinical oversight provided by a surgeon.

**Comment 2: Modeling of annotation variability**

The assumption that start and stop time variability can be approximated by two independent normal distributions (σ = 2 seconds) is reasonable for a methodological study. A brief clarification on how this value was estimated, or an explicit acknowledgment of this assumption as a limitation, could further improve transparency.

**Response 2**: We thank the Reviewer for this suggestion and agree that additional clarification improves transparency. In the revised manuscript, we now describe the internal annotation study conducted prior to this investigation (Line 142), in which multiple annotators independently annotated the same procedures. Using these replicate annotations, we estimated the variance of step start and stop times. We observed that annotation variability did not differ substantially across steps or procedure types. Based on these findings, we adopted a fixed standard deviation of 2 seconds and modeled start and stop times as independent normal distributions.

**Comment 3: Exclusion of multi-instance steps**

The exclusion of steps with multiple instances per case is logically justified to preserve the validity of the probabilistic framework. Please clarify:

- The proportion of steps or cases excluded due to this criterion.
- Whether excluded steps were evenly distributed across procedure types.

**Response 3:**

We thank the reviewer for this important request for clarification.

A total of 16124 annotated step instances were initially identified across all cases. Of these, 10393 step instances (64.46%) were excluded because they occurred multiple times within a case. Strictly speaking, our approach remains valid for multiple-instance steps provided the duration between instances is long relative to our start/stop uncertainty (on the order of several seconds). Only when the instances are in relative quick succession do our assumptions of independent start and stop times break down. However, rather than examine a mix of single- and multi-instance steps, we chose to exclude all of the later. We have qualified our statement in the manuscript explaining this exclusion.

The proportion of excluded multi-instance steps varied across procedure types, ranging from 34.6% to 78.4% of annotated steps per procedure. This variability reflects inherent differences in procedural workflow structure, as certain procedures contain more iterative or repeated steps by design. Despite this variation, a sufficient number of single-instance steps remained across all procedure types to support the proposed analysis.

We have revised the Results section to explicitly report the total number and proportion of excluded steps and to clarify their distribution across procedure types.

**Comment 4: Annotator characteristics**

Additional details regarding the annotators would strengthen the Methods section. Please consider specifying:

- The number of annotators involved.
- Their clinical background and level of training.

**Response 4:**

We thank the reviewer for this suggestion and have added additional details to the Methods section to clarify annotator characteristics (see Methods A. Data Collection paragraph 3).

As noted above, across procedure types, annotations were performed by teams of approximately 4–7 trained annotators during dataset construction. All annotators have backgrounds in biomedical engineering or were clinically trained medical officers with prior surgical experience. All annotators completed the same standardized training and calibration process prior to contributing annotations, as described in response to Comment 1.

**Comment 5: Discussion of limitations**

The Discussion is clear and appropriately balanced. It could be further strengthened by explicitly acknowledging:

- The fixed assumption of annotation variability (σ = 2 seconds) and the absence of a sensitivity analysis.
- Potential limitations in generalizing the approach to more iterative or complex surgical workflows.

**Response 5:**

We thank the reviewer for this thoughtful suggestion.

While this representative value, σ = 2 seconds, allowed us to illustrate the proposed probabilistic framework, we did not perform a formal sensitivity analysis to evaluate how varying σ would affect the results. Intuitively, however, decreasing the value would render the probabilistic OPIs more like the hard-boundary counterparts (which can be thought of as probabilistic OPIs with a delta function for the start/stop times), while increasing the value would “smooth” the OPI values, making them less sensitive to surgical activity during the start/stop times. Regardless, how these details would affect our results are not known. We have now clarified this assumption and its scope in the Discussion.

Second, regarding generalizability to more iterative or complex surgical workflows, the proposed framework remains applicable provided that annotation uncertainty does not systematically vary across repeated or iterative steps. The current formulation assumes stationary annotation uncertainty. When uncertainty itself varies across workflow phases or repetitions, extensions may be required. We have added text to the Discussion to clarify this point.

We sincerely thank the Reviewer for their constructive feedback and supportive evaluation. We believe the revisions have improved the clarity, transparency, and reproducibility of the manuscript.

Sincerely,

Ye
