# Supplementary material for: Validation of expected objective performance indicators in surgical data analysis
Source: PLoS One. 2026 Aug 7;21(8):e0355276. doi: 10.1371/journal.pone.0355276 (PMC13450760; doi:10.1371/journal.pone.0355276)
Supplement: Supplementary file 6 [file pone.0355276.s004.docx]

**Reviewer Comments**

The authors have adequately addressed the reviewer’s comments. The additional methodological clarifications have significantly improved the transparency and reproducibility of the study. The manuscript is now clear and suitable for publication.
