# Supplementary material for: Validation of expected objective performance indicators in surgical data analysis
Source: PLoS One. 2026 Aug 7;21(8):e0355276. doi: 10.1371/journal.pone.0355276 (PMC13450760; doi:10.1371/journal.pone.0355276)
Supplement: Supplementary file 5 [file pone.0355276.s002.docx]

**Reviewer Comments**

**General Assessment**

This manuscript presents a rigorous and well-executed methodological study addressing annotation-related variability in the computation of objective performance indicators (OPIs) in robotic surgery. The use of a large, multi-procedure dataset and a probabilistic framework to model uncertainty is a clear strength. The manuscript is well written and methodologically sound.

**Major Comments**

**1. Surgical step annotation framework**

The authors state that surgical steps were defined using an ontology **informed by consensus recommendations** (ref. 7). While this reference provides a **strong conceptual framework supporting structured, consensus-based step annotation,** additional methodological detail would improve **transparency and reproducibility.**

**Please clarify:**

- **How the step definitions were developed in practice following the principles outlined in the SAGES consensus.**
- **Whether the same step definitions were applied consistently across all cases within each procedure type.**
- **How annotators were trained and calibrated to apply these step definitions**.

**Minor Comments**

**2. Modeling of annotation variability**

The assumption that start and stop time variability can be approximated by two independent normal distributions (σ = 2 seconds) is reasonable for a methodological study. A brief clarification on how this value was estimated, or an explicit acknowledgment of this assumption as a limitation, could further improve transparency.

**3. Exclusion of multi-instance steps**

The exclusion of steps with multiple instances per case is logically justified to preserve the validity of the probabilistic framework. Please clarify:

- The proportion of steps or cases excluded due to this criterion.
- Whether excluded steps were evenly distributed across procedure types.

**4.Annotator characteristics**

**Additional details regarding the annotators would strengthen the Methods section.**

**Please consider specifying:**

- **The number of annotators involved.**
- **Their clinical background and level of training.**

**5. Discussion of limitations**

The Discussion is **clear and appropriately balanced**. It could be further strengthened by **explicitly acknowledging**:

- **The fixed assumption of annotation variability (σ = 2 seconds) and the absence of a sensitivity analysis.**
- **Potential limitations in generalizing the approach to more iterative or complex surgical workflows.**

**Summary Recommendation**

This is a strong methodological contribution. The suggested revisions are minor and primarily aim to improve methodological transparency.

*.*
