# Supplementary material for: Validation of expected objective performance indicators in surgical data analysis
Source: PLoS One. 2026 Aug 7;21(8):e0355276. doi: 10.1371/journal.pone.0355276 (PMC13450760; doi:10.1371/journal.pone.0355276)
Supplement: Supplementary file 7 [file pone.0355276.s006.docx]

Dear editor,

We would like to thank you for the consideration given to our manuscript and the reviewers' thoughtful assessment. With their feedback we have strengthened the paper with the broad readership of PLOS One in mind.

Respectfully,

Ye Li, Sreeram Kamabattula, Kiran Bhattacharryya, and Max Berniker

**Reviewer #1**

We thank the reviewer for their time and effort in reviewing our manuscript.

**Reviewer #2**

We thank the reviewer for their detailed review of our manuscript. We have carefully examined all comments and provide detailed point-by-point responses below. In cases where comments appeared to refer to the same underlying concern we grouped them together in our responses. Where appropriate, revisions have been incorporated into the manuscript and are identified in the corresponding responses. We think the modifications have improved the work’s presentation.

***Why is nominal OPI treated as ground truth?... You define nominal OPI as ground truth, but it is derived from noisy annotations. This is circular validation → you validate against your own noisy estimate. So no external or independent ground truth exists....***

We thank the reviewer for raising this important point. We agree that the nominal OPI is not absolute ground truth. Indeed, ground truth is not observable and can only be defined through consensus. However, our analysis was to assess the proposed probabilistic OPI method relative to the conventional approach, for a particular annotation. Ideally this reference annotation would be “ground truth.” We would argue though, that all we require is a reasonable reference annotation. To avoid this confusion, we have revised the manuscript to refer to the nominal OPI as a “reference” OPI rather than ground truth.

***You removed 64.46% of steps due to multi-instance cases. This is not a minor filtering step, it fundamentally biases the dataset. Real surgeries are iterative, your method ignores real-world complexity. So Your conclusions may not generalize at all.***

We appreciate the reviewer’s concern regarding the exclusion of multi-instance steps. To be clear, as was noted in the manuscript, this exclusion was a deliberate methodological decision rather than a filtering step intended to improve results.

To avoid potentially violating an assumption of the proposed probabilistic framework we restricted the study to steps with a single annotated instance per case. Note that we eliminated ~64% of the instances of steps, not the categories of steps, and even after that elimination we still have broad coverage of the surgical steps presented in SI Table 1. To your point that surgeries are iterative and complex, we note that multi-instance steps are often caused due to pauses in activity. As such a single step could be transformed into a two-instance step by merely pausing, not by performing some fundamentally distinct behavior. In this sense multi-instance steps are often merely single-instance steps back-to-back. As such we believe the results likely will generalize. However, we make no strict claims regarding multi-instance steps in general. Considering that single-instance steps do make up a significant portion of the clinical data, we see no reason why this finding is not valuable.

***How sensitive are results to σ ≠ 2 seconds? Fixed σ across all procedures and steps is not justified. Independence of start/stop times is clinically implausible. So no sensitivity analysis, results may be unstable ...Why assume independence of start and stop times?... Add sensitivity analysis for σ***

We thank the reviewer for these comments. As described in the Methods, the value of σ = 2 seconds was not arbitrarily selected, but was estimated from an internal annotation study. We did not find large variations across procedures. A formal sensitivity analysis of σ was not performed in this study. As noted in the Discussion, decreasing σ would make the probabilistic OPIs increasingly similar to their hard-boundary counterparts, whereas increasing σ would produce greater smoothing and reduce sensitivity to surgical activity near the annotated start and stop times. However, the quantitative effect of varying σ on the reported results was not evaluated and is beyond the scope of the present study but remains an area for future investigation.

The independence of start and stop times is a modeling assumption, and the reason we choose to eliminate the multi-instance steps from our analysis. Our general approach is to start with the simplest, most transparent model for analysis. Other than requiring stop times occur after start times, we did not see any reason to assume dependence. It is not clear to us why the reviewer thinks independence is implausible.

***What happens if σ varies across steps?***

We are not quite sure what the reviewer is asking. As discussed above, the framework itself is not restricted to a single uncertainty parameter and can accommodate alternative uncertainty models when such information is available. But as stated above, decreasing σ would make the probabilistic OPIs increasingly similar to their hard-boundary counterparts, whereas increasing σ would make the OPIs less sensitive to surgical activity near the annotation boundaries.

***Why exclude multi-instance steps instead of modeling them?***

Multi-instance steps were excluded because closely spaced repeated instances can violate the assumptions of the current probabilistic framework. The goal of this study was to validate the proposed method for single-instance steps. Extension to multi-instance workflows are beyond the scope of this study and planned for future work. We acknowledge this as an area for future investigation.

***Are results consistent per procedure type?***

Yes, the analyses were evaluated across procedure types and the overall findings were generally consistent. While a small number of procedure-specific comparisons did not reach statistical significance (Table 3, hand controller clutch rate), the overall trend remained consistent across procedure types and did not affect the conclusions of the study.

***How many annotators per case exactly?***

Following the standardized training and calibration process, each case was annotated by a single trained annotator. We have added this information to the Methods section to make the annotation process clearer.

***What is inter-annotator agreement (quantitatively)?***

With a single annotator per case no such analysis can be performed. In general, our annotators go through a lengthy training regimen wherein they complete a standardized training and calibration process with clinical oversight from a trained surgeon. Annotators do not work independently until their annotations are within five seconds of the surgeon reference annotations. We have clarified this process in the manuscript (3^rd^ paragraph of “Data Collection”).

***Why no correction for multiple hypothesis testing?***

The statistical tests in this study were performed as individual comparisons to evaluate specific aspects of the proposed methodology rather than as a large-scale hypothesis discovery exercise. In addition, the conclusions are supported by consistent trends across OPIs and procedure types, rather than by isolated statistically significant findings. Therefore, we did not apply a multiple-testing correction.

***Are distributions of OPIs normal (for t-tests validity)?***

The one-sample and two-sample t-tests in this study were applied to distributions generated from 500 resampled observations for each comparison. Given this sample size, the t-tests are generally robust to moderate departures from normality. In addition, for analyses where distributional assumptions were a greater concern (e.g., comparisons of normalized mean bias), we used the Wilcoxon signed-rank test rather than a parametric test. Therefore, separate normality assessments were not performed for each OPI distribution.

***Can you derive bias analytically under boundary uncertainty?***

No. The OPIs considered in this study are derived from time-series data and do not admit closed-form expressions. Investigation of analytical formulations for specific OPIs is an interesting direction for future work.

***Why not use a hierarchical Bayesian model for annotation uncertainty?***

There are of course many kinds of hierarchical models. As noted above, our general approach is to start with the simplest, most transparent model for our purposes. A hierarchical Bayesian framework could potentially model procedure-specific, step-specific, or annotator-specific uncertainty. The objective of our study was not to estimate the full structure of annotation uncertainty, but to evaluate whether incorporating uncertainty improves the robustness of OPI calculations.

***Why not model step boundaries as temporal point processes?***

Respectfully, we are not sure what the reviewer is suggesting. We could model the start and stop times are point processes to describe a set of start and stop annotations. But again, this was not the goal of our work and we adopted a simpler uncertainty model. Investigation of more complex temporal models remains an interesting direction for future work.

***Can uncertainty be learned from data instead of fixed σ?***

It is not obvious to us how this could be done. Since there are no ground truth annotations nor OPIs, they cannot be inferred from the data. Similarly, since each case had only a single annotator, we cannot directly measure it either. Ultimately though, this was beyond the scope of our study.

***How does your method compare to Monte Carlo integration?***

The proposed framework already uses repeated random sampling from the annotation uncertainty distributions. Specifically, start and stop times are repeatedly sampled, and the OPIs are recalculated for each sampled realization. The resulting distributions are then used to estimate expected OPIs and uncertainty.

***Why assume stationarity of annotation noise?***

It is not clear why annotation uncertainty would vary as a function of time. While we agree that it is likely to vary with other surgically relevant factors such as case complexity, we did not have access to that type of information. We acknowledge that annotation variability may differ under other annotation settings, and evaluation of non-stationary uncertainty models remains an area for future investigation.

***What is the identifiability of OPI under uncertain segmentation?***

We want to make sure there is no confusion. In our model, annotations are noisy, but fully observable. They are used to compute OPIs, which are fully observed as well. We are not inferring annotations or OPI values.

***Could this be framed as latent variable inference?***

We feel this may be related to the previous comment. The proposed framework is not formulated as a latent variable inference problem. Our objective is to incorporate uncertainty in the annotated start and stop times when calculating OPIs. This could be framed as an inference problem to estimate ground truth annotations, but more information beyond a single noisy annotation would be desired? Formulating the problem as a latent variable model would represent a different methodological framework and was beyond the scope of the present work.

***Tables are dense and hard to interpret***

We thank the reviewer for this feedback. The manuscript contains several tables summarizing results across multiple OPIs, procedure types, and statistical comparisons, which necessarily involve a substantial amount of information. We have reviewed the tables and believe their current organization and accompanying captions provide the information needed to interpret the results. As the reviewer did not identify a specific table or source of confusion, no changes were made.

***Better explain probabilistic intuition***

We appreciate the suggestion. The probabilistic OPI framework itself was introduced previously and is described in Reference [6], which is cited in the manuscript. The rationale for the probabilistic formulation is summarized in the Introduction and Methods, where annotation uncertainty in surgical step boundaries is incorporated into OPI calculations through probabilistic start and stop times. The primary objective of the present study is to validate the framework across a large clinical multi-procedure dataset rather than to introduce the methodology itself. As the reviewer did not identify a specific point requiring clarification, no changes were made.

**Reviewer #3**

We thank the reviewer for their time and effort in reviewing our manuscript.
