# Supplementary material for: Validation of expected objective performance indicators in surgical data analysis
Source: PLoS One. 2026 Aug 7;21(8):e0355276. doi: 10.1371/journal.pone.0355276 (PMC13450760; doi:10.1371/journal.pone.0355276)
Supplement: Peer review history

# Peer review: Decision Letter 0

**DOI:** 10.1371/journal.pone.0355276.r001

**Authors:** Daniel Parkes

12 Jan 2026

PONE-D-25-44565

Validation of Expected Objective Performance Indicators in Surgical Data Analysis

PLOS One

Dear Dr. Li,

Thank you for submitting your manuscript to PLOS ONE. After careful consideration, we feel that it has merit but does not fully meet PLOS ONE’s publication criteria as it currently stands. Therefore, we invite you to submit a revised version of the manuscript that addresses the points raised during the review process.

Please submit your revised manuscript by Feb 26 2026 11:59PM. If you will need more time than this to complete your revisions, please reply to this message or contact the journal office at plosone@plos.org. When you're ready to submit your revision, log on to https://www.editorialmanager.com/pone/ and select the 'Submissions Needing Revision' folder to locate your manuscript file.

Please include the following items when submitting your revised manuscript:

- A letter that responds to each point raised by the academic editor and reviewer(s). You should upload this letter as a separate file labeled 'Response to Reviewers'.
- A marked-up copy of your manuscript that highlights changes made to the original version. You should upload this as a separate file labeled 'Revised Manuscript with Track Changes'.
- An unmarked version of your revised paper without tracked changes. You should upload this as a separate file labeled 'Manuscript'.

If you would like to make changes to your financial disclosure, please include your updated statement in your cover letter. Guidelines for resubmitting your figure files are available below the reviewer comments at the end of this letter.

If applicable, we recommend that you deposit your laboratory protocols in protocols.io to enhance the reproducibility of your results. Protocols.io assigns your protocol its own identifier (DOI) so that it can be cited independently in the future. For instructions see: https://journals.plos.org/plosone/s/submission-guidelines#loc-laboratory-protocols. Additionally, PLOS ONE offers an option for publishing peer-reviewed Lab Protocol articles, which describe protocols hosted on protocols.io. Read more information on sharing protocols at https://plos.org/protocols?utm_medium=editorial-email&utm_source=authorletters&utm_campaign=protocols.

We look forward to receiving your revised manuscript.

Kind regards,

Daniel Parkes, PhD

Staff Editor

PLOS One

Journal Requirements:

When submitting your revision, we need you to address these additional requirements.

1. Please ensure that your manuscript meets PLOS ONE's style requirements, including those for file naming. The PLOS ONE style templates can be found at

https://journals.plos.org/plosone/s/file?id=wjVg/PLOSOne_formatting_sample_main_body.pdf and

https://journals.plos.org/plosone/s/file?id=ba62/PLOSOne_formatting_sample_title_authors_affiliations.pdf

2. When completing the data availability statement of the submission form, you indicated that you will make your data available on acceptance. We strongly recommend all authors decide on a data sharing plan before acceptance, as the process can be lengthy and hold up publication timelines. Please note that, though access restrictions are acceptable now, your entire data will need to be made freely accessible if your manuscript is accepted for publication. This policy applies to all data except where public deposition would breach compliance with the protocol approved by your research ethics board. If you are unable to adhere to our open data policy, please kindly revise your statement to explain your reasoning and we will seek the editor's input on an exemption. Please be assured that, once you have provided your new statement, the assessment of your exemption will not hold up the peer review process.

3. If the reviewer comments include a recommendation to cite specific previously published works, please review and evaluate these publications to determine whether they are relevant and should be cited. There is no requirement to cite these works unless the editor has indicated otherwise.

[Note: HTML markup is below. Please do not edit.]

Reviewers' comments:

Reviewer's Responses to Questions

**Comments to the Author**

1. Is the manuscript technically sound, and do the data support the conclusions?

The manuscript must describe a technically sound piece of scientific research with data that supports the conclusions. Experiments must have been conducted rigorously, with appropriate controls, replication, and sample sizes. The conclusions must be drawn appropriately based on the data presented.

Reviewer #1: Yes

**********

2. Has the statistical analysis been performed appropriately and rigorously?

Reviewer #1: Yes

**********

3. Have the authors made all data underlying the findings in their manuscript fully available?

The PLOS Data policy requires authors to make all data underlying the findings described in their manuscript fully available without restriction, with rare exception (please refer to the Data Availability Statement in the manuscript PDF file). The data should be provided as part of the manuscript or its supporting information, or deposited to a public repository. For example, in addition to summary statistics, the data points behind means, medians and variance measures should be available. If there are restrictions on publicly sharing data—e.g. participant privacy or use of data from a third party—those must be specified.

Reviewer #1: Yes

**********

4. Is the manuscript presented in an intelligible fashion and written in standard English?

PLOS ONE does not copyedit accepted manuscripts, so the language in submitted articles must be clear, correct, and unambiguous. Any typographical or grammatical errors should be corrected at revision, so please note any specific errors here.

Reviewer #1: Yes

**********

5. Review Comments to the Author

Please use the space provided to explain your answers to the questions above. You may also include additional comments for the author, including concerns about dual publication, research ethics, or publication ethics. (Please upload your review as an attachment if it exceeds 20,000 characters)

Reviewer #1: Reviewer Comments

General Assessment

This manuscript presents a rigorous and well-executed methodological study addressing annotation-related variability in the computation of objective performance indicators (OPIs) in robotic surgery. The use of a large, multi-procedure dataset and a probabilistic framework to model uncertainty is a clear strength. The manuscript is well written and methodologically sound.

Major Comments

1. Surgical step annotation framework

The authors state that surgical steps were defined using an ontology informed by consensus recommendations (ref. 7). While this reference provides a strong conceptual framework supporting structured, consensus-based step annotation, additional methodological detail would improve transparency and reproducibility.

Please clarify:

• How the step definitions were developed in practice following the principles outlined in the SAGES consensus.

• Whether the same step definitions were applied consistently across all cases within each procedure type.

• How annotators were trained and calibrated to apply these step definitions.

Minor Comments

2. Modeling of annotation variability

The assumption that start and stop time variability can be approximated by two independent normal distributions (σ = 2 seconds) is reasonable for a methodological study. A brief clarification on how this value was estimated, or an explicit acknowledgment of this assumption as a limitation, could further improve transparency.

3. Exclusion of multi-instance steps

The exclusion of steps with multiple instances per case is logically justified to preserve the validity of the probabilistic framework. Please clarify:

• The proportion of steps or cases excluded due to this criterion.

• Whether excluded steps were evenly distributed across procedure types.

4.Annotator characteristics

Additional details regarding the annotators would strengthen the Methods section.

Please consider specifying:

• The number of annotators involved.

• Their clinical background and level of training.

5. Discussion of limitations

The Discussion is clear and appropriately balanced. It could be further strengthened by explicitly acknowledging:

• The fixed assumption of annotation variability (σ = 2 seconds) and the absence of a sensitivity analysis.

• Potential limitations in generalizing the approach to more iterative or complex surgical workflows.

Summary Recommendation

This is a strong methodological contribution. The suggested revisions are minor and primarily aim to improve methodological transparency.

**********

6. PLOS authors have the option to publish the peer review history of their article (what does this mean?). If published, this will include your full peer review and any attached files.

If you choose “no”, your identity will remain anonymous but your review may still be made public.

**Do you want your identity to be public for this peer review?**  For information about this choice, including consent withdrawal, please see our Privacy Policy.

Reviewer #1: No

**********

[NOTE: If reviewer comments were submitted as an attachment file, they will be attached to this email and accessible via the submission site. Please log into your account, locate the manuscript record, and check for the action link "View Attachments". If this link does not appear, there are no attachment files.]

To ensure your figures meet our technical requirements, please review our figure guidelines: https://journals.plos.org/plosone/s/figures

You may also use PLOS’s free figure tool, NAAS, to help you prepare publication quality figures: https://journals.plos.org/plosone/s/figures#loc-tools-for-figure-preparation.

NAAS will assess whether your figures meet our technical requirements by comparing each figure against our figure specifications.

Attachment Submitted filename: Reviewer Comments 2026.docx

# Author comment: Author response to Decision Letter 1

**DOI:** 10.1371/journal.pone.0355276.r002

11 Mar 2026

Dear reviewer,

We thank the Reviewer for their thoughtful and constructive evaluation of our manuscript. Below, we provide point-by-point responses to all comments. All changes have been incorporated into the revised manuscript, with corresponding sections indicated.

Comment 1: Surgical step annotation framework

The authors state that surgical steps were defined using an ontology informed by consensus recommendations (ref. 7). While this reference provides a strong conceptual framework supporting structured, consensus-based step annotation, additional methodological detail would improve transparency and reproducibility.

Please clarify:

How the step definitions were developed in practice following the principles outlined in the SAGES consensus.

Whether the same step definitions were applied consistently across all cases within each procedure type.

How annotators were trained and calibrated to apply these step definitions.

Response 1: We thank the reviewer for raising these important topics. We have revised the manuscript accordingly to provide the additional methodological detail. Our responses to the specific points are provided below.

1. To improve clarity, we have added a citation to a recently published paper from our annotation team that provides a detailed description of how surgical step annotations are defined, operationalized, and applied in practice within our annotation workflow. This paper describes the construction and use of step-level annotation ontologies, the annotation process, and quality control procedures, and is now cited in the revised manuscript (see Method A. Data Collection paragraph 2).

2. Yes, for the majority of cases, the same step definitions were applied consistently. However, for a few procedure types, small adjustments to the start and stop parameters of a step (though not the definition of the step) were introduced early in the dataset construction, to improve inter-annotator alignment. From that point forward, all annotations were consistently applied to cases of that procedure type.

3. Annotators underwent a structured training and calibration process prior to contributing annotations. Training typically consisted of annotating 10–20 representative cases of the same procedure type, spanning a range of case complexity and approaches. During this phase, each trainee’s annotations were compared against a “gold-standard” reference produced by the most experienced annotator for that procedure, under surgeon oversight.

Calibration criteria required temporal alignment of step start and end times within approximately 5 seconds of the gold-standard annotations. Trainees received iterative feedback and were only authorized to annotate independently after achieving this alignment criterion. Annotators who did not meet the required agreement threshold continued training until alignment was achieved or were not assigned to that procedure type.

In addition to initial training, ongoing quality control was performed through periodic consensus reviews (e.g., quarterly or bi-quarterly), during which inter-annotator alignment was reassessed to ensure long-term consistency. All annotators followed the same training and calibration protocol, and annotation teams for each procedure typically consisted of approximately 4–7 trained annotators, with overall clinical oversight provided by a surgeon.

Comment 2: Modeling of annotation variability

The assumption that start and stop time variability can be approximated by two independent normal distributions (σ = 2 seconds) is reasonable for a methodological study. A brief clarification on how this value was estimated, or an explicit acknowledgment of this assumption as a limitation, could further improve transparency.

Response 2: We thank the Reviewer for this suggestion and agree that additional clarification improves transparency. In the revised manuscript, we now describe the internal annotation study conducted prior to this investigation (Line 142), in which multiple annotators independently annotated the same procedures. Using these replicate annotations, we estimated the variance of step start and stop times. We observed that annotation variability did not differ substantially across steps or procedure types. Based on these findings, we adopted a fixed standard deviation of 2 seconds and modeled start and stop times as independent normal distributions.

Comment 3: Exclusion of multi-instance steps

The exclusion of steps with multiple instances per case is logically justified to preserve the validity of the probabilistic framework. Please clarify:

The proportion of steps or cases excluded due to this criterion.

Whether excluded steps were evenly distributed across procedure types.

Response 3:

We thank the reviewer for this important request for clarification.

A total of 16124 annotated step instances were initially identified across all cases. Of these, 10393 step instances (64.46%) were excluded because they occurred multiple times within a case. Strictly speaking, our approach remains valid for multiple-instance steps provided the duration between instances is long relative to our start/stop uncertainty (on the order of several seconds). Only when the instances are in relative quick succession do our assumptions of independent start and stop times break down. However, rather than examine a mix of single- and multi-instance steps, we chose to exclude all of the later. We have qualified our statement in the manuscript explaining this exclusion.

The proportion of excluded multi-instance steps varied across procedure types, ranging from 34.6% to 78.4% of annotated steps per procedure. This variability reflects inherent differences in procedural workflow structure, as certain procedures contain more iterative or repeated steps by design. Despite this variation, a sufficient number of single-instance steps remained across all procedure types to support the proposed analysis.

We have revised the Results section to explicitly report the total number and proportion of excluded steps and to clarify their distribution across procedure types.

Comment 4: Annotator characteristics

Additional details regarding the annotators would strengthen the Methods section. Please consider specifying:

The number of annotators involved.

Their clinical background and level of training.

Response 4:

We thank the reviewer for this suggestion and have added additional details to the Methods section to clarify annotator characteristics (see Methods A. Data Collection paragraph 3).

As noted above, across procedure types, annotations were performed by teams of approximately 4–7 trained annotators during dataset construction. All annotators have backgrounds in biomedical engineering or were clinically trained medical officers with prior surgical experience. All annotators completed the same standardized training and calibration process prior to contributing annotations, as described in response to Comment 1.

Comment 5: Discussion of limitations

The Discussion is clear and appropriately balanced. It could be further strengthened by explicitly acknowledging:

The fixed assumption of annotation variability (σ = 2 seconds) and the absence of a sensitivity analysis.

Potential limitations in generalizing the approach to more iterative or complex surgical workflows.

Response 5:

We thank the reviewer for this thoughtful suggestion.

While this representative value, σ = 2 seconds, allowed us to illustrate the proposed probabilistic framework, we did not perform a formal sensitivity analysis to evaluate how varying σ would affect the results. Intuitively, however, decreasing the value would render the probabilistic OPIs more like the hard-boundary counterparts (which can be thought of as probabilistic OPIs with a delta function for the start/stop times), while increasing the value would “smooth” the OPI values, making them less sensitive to surgical activity during the start/stop times. Regardless, how these details would affect our results are not known. We have now clarified this assumption and its scope in the Discussion.

Second, regarding generalizability to more iterative or complex surgical workflows, the proposed framework remains applicable provided that annotation uncertainty does not systematically vary across repeated or iterative steps. The current formulation assumes stationary annotation uncertainty. When uncertainty itself varies across workflow phases or repetitions, extensions may be required. We have added text to the Discussion to clarify this point.

We sincerely thank the Reviewer for their constructive feedback and supportive evaluation. We believe the revisions have improved the clarity, transparency, and reproducibility of the manuscript.

Sincerely,

Ye

Attachment Submitted filename: Response to Reviewers.docx

# Peer review: Decision Letter 1

**DOI:** 10.1371/journal.pone.0355276.r003

**Authors:** Hongyang Ma

30 Apr 2026

PONE-D-25-44565R1

Validation of expected objective performance indicators in surgical data analysis

PLOS One

Dear Dr. Li,

Thank you for submitting your manuscript to PLOS ONE. After careful consideration, we feel that it has merit but does not fully meet PLOS ONE’s publication criteria as it currently stands. Therefore, we invite you to submit a revised version of the manuscript that addresses the points raised during the review process.

Please submit your revised manuscript by Jun 14 2026 11:59PM. If you will need more time than this to complete your revisions, please reply to this message or contact the journal office at plosone@plos.org. When you're ready to submit your revision, log on to https://www.editorialmanager.com/pone/ and select the 'Submissions Needing Revision' folder to locate your manuscript file.

Please include the following items when submitting your revised manuscript:

- A letter that responds to each point raised by the academic editor and reviewer(s). You should upload this letter as a separate file labeled 'Response to Reviewers'.
- A marked-up copy of your manuscript that highlights changes made to the original version. You should upload this as a separate file labeled 'Revised Manuscript with Track Changes'.
- An unmarked version of your revised paper without tracked changes. You should upload this as a separate file labeled 'Manuscript'.

If you would like to make changes to your financial disclosure, please include your updated statement in your cover letter. Guidelines for resubmitting your figure files are available below the reviewer comments at the end of this letter.

If applicable, we recommend that you deposit your laboratory protocols in protocols.io to enhance the reproducibility of your results. Protocols.io assigns your protocol its own identifier (DOI) so that it can be cited independently in the future. For instructions see: https://journals.plos.org/plosone/s/submission-guidelines#loc-laboratory-protocols. Additionally, PLOS ONE offers an option for publishing peer-reviewed Lab Protocol articles, which describe protocols hosted on protocols.io. Read more information on sharing protocols at https://plos.org/protocols?utm_medium=editorial-email&utm_source=authorletters&utm_campaign=protocols.

As the corresponding author, your ORCID iD is verified in the submission system and will appear in the published article. PLOS supports the use of ORCID, and we encourage all coauthors to register for an ORCID iD and use it as well. Please encourage your coauthors to verify their ORCID iD within the submission system before final acceptance, as unverified ORCID iDs will not appear in the published article. *Only*  the individual author can complete the verification step; PLOS staff *cannot*  verify ORCID iDs on behalf of authors.

We look forward to receiving your revised manuscript.

Kind regards,

Hongyang Ma, Ph.D., D.D.S

Guest Editor

PLOS One

Reviewers' comments:

Reviewer's Responses to Questions

**Comments to the Author**

1. If the authors have adequately addressed your comments raised in a previous round of review and you feel that this manuscript is now acceptable for publication, you may indicate that here to bypass the “Comments to the Author” section, enter your conflict of interest statement in the “Confidential to Editor” section, and submit your "Accept" recommendation.

Reviewer #1: All comments have been addressed

Reviewer #2: (No Response)

Reviewer #3: All comments have been addressed

**********

2. Is the manuscript technically sound, and do the data support the conclusions?

The manuscript must describe a technically sound piece of scientific research with data that supports the conclusions. Experiments must have been conducted rigorously, with appropriate controls, replication, and sample sizes. The conclusions must be drawn appropriately based on the data presented.

Reviewer #1: Yes

Reviewer #2: (No Response)

Reviewer #3: Yes

**********

3. Has the statistical analysis been performed appropriately and rigorously?

Reviewer #1: Yes

Reviewer #2: (No Response)

Reviewer #3: Yes

**********

4. Have the authors made all data underlying the findings in their manuscript fully available?

The PLOS Data policy requires authors to make all data underlying the findings described in their manuscript fully available without restriction, with rare exception (please refer to the Data Availability Statement in the manuscript PDF file). The data should be provided as part of the manuscript or its supporting information, or deposited to a public repository. For example, in addition to summary statistics, the data points behind means, medians and variance measures should be available. If there are restrictions on publicly sharing data—e.g. participant privacy or use of data from a third party—those must be specified.

Reviewer #1: No

Reviewer #2: (No Response)

Reviewer #3: Yes

**********

5. Is the manuscript presented in an intelligible fashion and written in standard English?

PLOS ONE does not copyedit accepted manuscripts, so the language in submitted articles must be clear, correct, and unambiguous. Any typographical or grammatical errors should be corrected at revision, so please note any specific errors here.

Reviewer #1: Yes

Reviewer #2: (No Response)

Reviewer #3: Yes

**********

6. Review Comments to the Author

Please use the space provided to explain your answers to the questions above. You may also include additional comments for the author, including concerns about dual publication, research ethics, or publication ethics. (Please upload your review as an attachment if it exceeds 20,000 characters)

Reviewer #1: The authors have adequately addressed the reviewer’s comments. The additional methodological clarifications have significantly improved the transparency and reproducibility of the study. The manuscript is now clear and suitable for publication

Reviewer #2: This study proposes and validates a probabilistic framework for computing Objective Performance Indicators (OPIs) in robotic-assisted surgery, aiming to mitigate variability arising from ambiguous surgical step annotations. Using a large dataset of 1,016 surgical cases and 5,408 annotated steps, the authors compare the proposed “expected OPI” method against conventional hard-boundary OPIs. Through resampling-based statistical analysis, they show that the probabilistic approach yields lower bias and variance under annotation uncertainty, suggesting improved robustness and reliability for surgical performance assessment. Major revision (borderline reject if not improved).

Why is nominal OPI treated as ground truth?

You removed 64.46% of steps due to multi-instance cases.

This is not a minor filtering step, it fundamentally biases the dataset.

Real surgeries are iterative, your method ignores real-world complexity. So Your conclusions may not generalize at all.

You define nominal OPI as ground truth, but it is derived from noisy annotations. This is circular validation → you validate against your own noisy estimate. So no external or independent ground truth exists.

How sensitive are results to σ ≠ 2 seconds?

Fixed σ across all procedures and steps is not justified. Independence of start/stop times is clinically implausible. So no sensitivity analysis, results may be unstable.

Why assume independence of start and stop times?

What happens if σ varies across steps?

Why exclude multi-instance steps instead of modeling them?

Are results consistent per procedure type?

How many annotators per case exactly?

What is inter-annotator agreement (quantitatively)?

Why no correction for multiple hypothesis testing?

Are distributions of OPIs normal (for t-tests validity)?

Can you derive bias analytically under boundary uncertainty?

Why not use a hierarchical Bayesian model for annotation uncertainty?

Why not model step boundaries as temporal point processes?

Can uncertainty be learned from data instead of fixed σ?

How does your method compare to Monte Carlo integration?

Why assume stationarity of annotation noise?

What is the identifiability of OPI under uncertain segmentation?

Could this be framed as latent variable inference?

Tables are dense and hard to interpret

Better explain probabilistic intuition

Add sensitivity analysis for σ

Reviewer #3: I love the topic and the ingenuity behind it. While reproducibility might be a bit difficult, the rationale behind the topic is clear and the work was thorough. All corrections previously outlined has been worked on and put in place. Well done.

**********

7. PLOS authors have the option to publish the peer review history of their article (what does this mean?). If published, this will include your full peer review and any attached files.

If you choose “no”, your identity will remain anonymous but your review may still be made public.

**Do you want your identity to be public for this peer review?**  For information about this choice, including consent withdrawal, please see our Privacy Policy.

Reviewer #1: No

Reviewer #2: No

Reviewer #3: **Yes:** ADEFUSI TEMILOLUWA

**********

[NOTE: If reviewer comments were submitted as an attachment file, they will be attached to this email and accessible via the submission site. Please log into your account, locate the manuscript record, and check for the action link "View Attachments". If this link does not appear, there are no attachment files.]

To ensure your figures meet our technical requirements, please review our figure guidelines: https://journals.plos.org/plosone/s/figures

You may also use PLOS’s free figure tool, NAAS, to help you prepare publication quality figures: https://journals.plos.org/plosone/s/figures#loc-tools-for-figure-preparation.

NAAS will assess whether your figures meet our technical requirements by comparing each figure against our figure specifications.

Attachment Submitted filename: Reviewer Comments 2026 2.docx

# Author comment: Author response to Decision Letter 2

**DOI:** 10.1371/journal.pone.0355276.r004

6 Jul 2026

Dear editor,

We would like to thank you for the consideration given to our manuscript and the reviewers' thoughtful assessment. With their feedback we have strengthened the paper with the broad readership of PLOS One in mind.

Respectfully,

Ye Li, Sreeram Kamabattula, Kiran Bhattacharryya, and Max Berniker

Reviewer #1

We thank the reviewer for their time and effort in reviewing our manuscript.

Reviewer #2

We thank the reviewer for their detailed review of our manuscript. We have carefully examined all comments and provide detailed point-by-point responses below. In cases where comments appeared to refer to the same underlying concern we grouped them together in our responses. Where appropriate, revisions have been incorporated into the manuscript and are identified in the corresponding responses. We think the modifications have improved the work’s presentation.

Comment: Why is nominal OPI treated as ground truth?... You define nominal OPI as ground truth, but it is derived from noisy annotations. This is circular validation → you validate against your own noisy estimate. So no external or independent ground truth exists....

Response: We thank the reviewer for raising this important point. We agree that the nominal OPI is not absolute ground truth. Indeed, ground truth is not observable and can only be defined through consensus. However, our analysis was to assess the proposed probabilistic OPI method relative to the conventional approach, for a particular annotation. Ideally this reference annotation would be “ground truth.” We would argue though, that all we require is a reasonable reference annotation. To avoid this confusion, we have revised the manuscript to refer to the nominal OPI as a “reference” OPI rather than ground truth.

Comment: You removed 64.46% of steps due to multi-instance cases. This is not a minor filtering step, it fundamentally biases the dataset. Real surgeries are iterative, your method ignores real-world complexity. So Your conclusions may not generalize at all.

Response: We appreciate the reviewer’s concern regarding the exclusion of multi-instance steps. To be clear, as was noted in the manuscript, this exclusion was a deliberate methodological decision rather than a filtering step intended to improve results.

To avoid potentially violating an assumption of the proposed probabilistic framework we restricted the study to steps with a single annotated instance per case. Note that we eliminated ~64% of the instances of steps, not the categories of steps, and even after that elimination we still have broad coverage of the surgical steps presented in SI Table 1. To your point that surgeries are iterative and complex, we note that multi-instance steps are often caused due to pauses in activity. As such a single step could be transformed into a two-instance step by merely pausing, not by performing some fundamentally distinct behavior. In this sense multi-instance steps are often merely single-instance steps back-to-back. As such we believe the results likely will generalize. However, we make no strict claims regarding multi-instance steps in general. Considering that single-instance steps do make up a significant portion of the clinical data, we see no reason why this finding is not valuable.

Comment: How sensitive are results to σ ≠ 2 seconds? Fixed σ across all procedures and steps is not justified. Independence of start/stop times is clinically implausible. So no sensitivity analysis, results may be unstable ...Why assume independence of start and stop times?... Add sensitivity analysis for σ

Response: We thank the reviewer for these comments. As described in the Methods, the value of σ = 2 seconds was not arbitrarily selected, but was estimated from an internal annotation study. We did not find large variations across procedures. A formal sensitivity analysis of σ was not performed in this study. As noted in the Discussion, decreasing σ would make the probabilistic OPIs increasingly similar to their hard-boundary counterparts, whereas increasing σ would produce greater smoothing and reduce sensitivity to surgical activity near the annotated start and stop times. However, the quantitative effect of varying σ on the reported results was not evaluated and is beyond the scope of the present study but remains an area for future investigation.

The independence of start and stop times is a modeling assumption, and the reason we choose to eliminate the multi-instance steps from our analysis. Our general approach is to start with the simplest, most transparent model for analysis. Other than requiring stop times occur after start times, we did not see any reason to assume dependence. It is not clear to us why the reviewer thinks independence is implausible.

Comment: What happens if σ varies across steps?

Response: We are not quite sure what the reviewer is asking. As discussed above, the framework itself is not restricted to a single uncertainty parameter and can accommodate alternative uncertainty models when such information is available. But as stated above, decreasing σ would make the probabilistic OPIs increasingly similar to their hard-boundary counterparts, whereas increasing σ would make the OPIs less sensitive to surgical activity near the annotation boundaries.

Comment: Why exclude multi-instance steps instead of modeling them?

Response: Multi-instance steps were excluded because closely spaced repeated instances can violate the assumptions of the current probabilistic framework. The goal of this study was to validate the proposed method for single-instance steps. Extension to multi-instance workflows are beyond the scope of this study and planned for future work. We acknowledge this as an area for future investigation.

Comment: Are results consistent per procedure type?

Response: Yes, the analyses were evaluated across procedure types and the overall findings were generally consistent. While a small number of procedure-specific comparisons did not reach statistical significance (Table 3, hand controller clutch rate), the overall trend remained consistent across procedure types and did not affect the conclusions of the study.

Comment: How many annotators per case exactly?

Response: Following the standardized training and calibration process, each case was annotated by a single trained annotator. We have added this information to the Methods section to make the annotation process clearer.

Comment: What is inter-annotator agreement (quantitatively)?

Response: With a single annotator per case no such analysis can be performed. In general, our annotators go through a lengthy training regimen wherein they complete a standardized training and calibration process with clinical oversight from a trained surgeon. Annotators do not work independently until their annotations are within five seconds of the surgeon reference annotations. We have clarified this process in the manuscript (3rd paragraph of “Data Collection”).

Comment: Why no correction for multiple hypothesis testing?

Response: The statistical tests in this study were performed as individual comparisons to evaluate specific aspects of the proposed methodology rather than as a large-scale hypothesis discovery exercise. In addition, the conclusions are supported by consistent trends across OPIs and procedure types, rather than by isolated statistically significant findings. Therefore, we did not apply a multiple-testing correction.

Comment: Are distributions of OPIs normal (for t-tests validity)?

Response: The one-sample and two-sample t-tests in this study were applied to distributions generated from 500 resampled observations for each comparison. Given this sample size, the t-tests are generally robust to moderate departures from normality. In addition, for analyses where distributional assumptions were a greater concern (e.g., comparisons of normalized mean bias), we used the Wilcoxon signed-rank test rather than a parametric test. Therefore, separate normality assessments were not performed for each OPI distribution.

Comment: Can you derive bias analytically under boundary uncertainty?

Response: No. The OPIs considered in this study are derived from time-series data and do not admit closed-form expressions. Investigation of analytical formulations for specific OPIs is an interesting direction for future work.

Comment: Why not use a hierarchical Bayesian model for annotation uncertainty?

Response: There are of course many kinds of hierarchical models. As noted above, our general approach is to start with the simplest, most transparent model for our purposes. A hierarchical Bayesian framework could potentially model procedure-specific, step-specific, or annotator-specific uncertainty. The objective of our study was not to estimate the full structure of annotation uncertainty, but to evaluate whether incorporating uncertainty improves the robustness of OPI calculations.

Comment: Why not model step boundaries as temporal point processes?

Response: Respectfully, we are not sure what the reviewer is suggesting. We could model the start and stop times are point processes to describe a set of start and stop annotations. But again, this was not the goal of our work and we adopted a simpler uncertainty model. Investigation of more complex temporal models remains an interesting direction for future work.

Comment: Can uncertainty be learned from data instead of fixed σ?

Response: It is not obvious to us how this could be done. Since there are no ground truth annotations nor OPIs, they cannot be inferred from the data. Similarly, since each case had only a single annotator, we cannot directly measure it either. Ultimately though, this was beyond the scope of our study.

Comment: How does your method compare to Monte Carlo integration?

Response: The proposed framework already uses repeated random sampling from the annotation uncertainty distributions. Specifically, start and stop times are repeatedly sampled, and the OPIs are recalculated for each sampled realization. The resulting distributions are then used to estimate expected OPIs and uncertainty.

Comment: Why assume stationarity of annotation noise?

Response: It is not clear why annotation uncertainty would vary as a function of time. While we agree that it is likely to vary with other surgically relevant factors such as case complexity, we did not have access to that type of information. We acknowledge that annotation variability may differ under other annotation settings, and evaluation of non-stationary uncertainty models remains an area for future investigation.

Comment: What is the identifiability of OPI under uncertain segmentation?

Response: We want to make sure there is no confusion. In our model, annotations are noisy, but fully observable. They are used to compute OPIs, which are fully observed as well. We are not inferring annotations or OPI values.

Comment: Could this be framed as latent variable inference?

Response: We feel this may be related to the previous comment. The proposed framework is not formulated as a latent variable inference problem. Our objective is to incorporate uncertainty in the annotated start and stop times when calculating OPIs. This could be framed as an inference problem to estimate ground truth annotations, but more information beyond a single noisy annotation would be desired? Formulating the problem as a latent variable model would represent a different methodological framework and was beyond the scope of the present work.

Comment: Tables are dense and hard to interpret

Response: We thank the reviewer for this feedback. The manuscript contains several tables summarizing results across multiple OPIs, procedure types, and statistical comparisons, which necessarily involve a substantial amount of information. We have reviewed the tables and believe their current organization and accompanying captions provide the information needed to interpret the results. As the reviewer did not identify a specific table or source of confusion, no changes were made.

Comment: Better explain probabilistic intuition

Response: We appreciate the suggestion. The probabilistic OPI framework itself was introduced previously and is described in Reference [6], which is cited in the manuscript. The rationale for the probabilistic formulation is summarized in the Introduction and Methods, where annotation uncertainty in surgical step boundaries is incorporated into OPI calculations through probabilistic start and stop times. The primary objective of the present study is to validate the framework across a large clinical multi-procedure dataset rather than to introduce the methodology itself. As the reviewer did not identify a specific point requiring clarification, no changes were made.

Reviewer #3

We thank the reviewer for their time and effort in reviewing our manuscript.

Attachment Submitted filename: Response_to_Reviewers_auresp_2.docx

# Editor report: Decision Letter 2

**DOI:** 10.1371/journal.pone.0355276.r005

**Authors:** Hongyang Ma

20 Jul 2026

Validation of expected objective performance indicators in surgical data analysis

PONE-D-25-44565R2

Dear Dr. Li,

We’re pleased to inform you that your manuscript has been judged scientifically suitable for publication and will be formally accepted for publication once it meets all outstanding technical requirements.

Within one week, you’ll receive an e-mail detailing the required amendments. When these have been addressed, you’ll receive a formal acceptance letter and your manuscript will be scheduled for publication.

An invoice will be generated when your article is formally accepted. Please note, if your institution has a publishing partnership with PLOS and your article meets the relevant criteria, all or part of your publication costs will be covered. Please make sure your user information is up-to-date by logging into Editorial Manager at Editorial Manager® and clicking the ‘Update My Information' link at the top of the page. For questions related to billing, please contact billing support.

If your institution or institutions have a press office, please notify them about your upcoming paper to help maximize its impact. If they’ll be preparing press materials, please inform our press team as soon as possible -- no later than 48 hours after receiving the formal acceptance. Your manuscript will remain under strict press embargo until 2 pm Eastern Time on the date of publication. For more information, please contact onepress@plos.org.

Kind regards,

Hongyang Ma, Ph.D., D.D.S

Guest Editor

PLOS One

Additional Editor Comments (optional):

Reviewers' comments:

# Editor report: Acceptance letter

**DOI:** 10.1371/journal.pone.0355276.r006

**Authors:** Hongyang Ma

PONE-D-25-44565R2

PLOS One

Dear Dr. Li,

I'm pleased to inform you that your manuscript has been deemed suitable for publication in PLOS One. Congratulations! Your manuscript is now being handed over to our production team.

At this stage, our production department will prepare your paper for publication. This includes ensuring the following:

* All references, tables, and figures are properly cited

* All relevant supporting information is included in the manuscript submission,

* There are no issues that prevent the paper from being properly typeset

You will receive further instructions from the production team, including instructions on how to review your proof when it is ready. Please keep in mind that we are working through a large volume of accepted articles, so please give us a few days to review your paper and let you know the next and final steps.

Lastly, if your institution or institutions have a press office, please let them know about your upcoming paper now to help maximize its impact. If they'll be preparing press materials, please inform our press team within the next 48 hours. Your manuscript will remain under strict press embargo until 2 pm Eastern Time on the date of publication. For more information, please contact onepress@plos.org.

You will receive an invoice from PLOS for your publication fee after your manuscript has reached the completed accept phase. If you receive an email requesting payment before acceptance or for any other service, this may be a phishing scheme. Learn how to identify phishing emails and protect your accounts at https://explore.plos.org/phishing.

If we can help with anything else, please email us at customercare@plos.org.

Thank you for submitting your work to PLOS ONE and supporting open access.

Kind regards,

PLOS ONE Editorial Office Staff

on behalf of

Dr. Hongyang Ma

Guest Editor

PLOS One
